# VISIONARA – developing a concept for decentralized competency-based learning in primary health care and its proposed evaluation

**DOI:** 10.3205/zma001874

**Published:** 2026-09-15

**Authors:** Judith Lübbert, Dorothee Schüle, Cornelia Wachter, Sonia Kurczyk, Attila Altiner, Svetla Loukanova, Rüdiger Leutgeb

**Affiliations:** 1 Heidelberg University Hospital, Department of General Practice and Health Services Research, Heidelberg, Germany

**Keywords:** undergraduate medical education, primary health care, competency-based education, general practice education, family practice education, clinical clerkship, curriculum, teaching

## Abstract

**Background:**

To educate high-quality medical graduates who can contribute to a resilient, primary care-based health system, medical education must shift its focus towards decentralized and curricular competency-based medical education (CBME). However, undergraduate general practice education in Germany remains fragmented: theoretical content is delivered at universities, while practical experience is confined to clerkships in community practices, which often lack sufficient didactic infrastructure. This persistent separation of theory and practice hinders the genuine transformation towards CBME. New models that embed structured teaching directly within real care environments are required to bridge this gap.

**Approach:**

VISIOn Network of Academic Reference Practices in General Medicine (VISIONARA) introduces a novel model for decentralized competency-oriented education in general practice. By establishing academic teaching clusters within community practices, the program creates structured, contextually anchored learning environments that enable experiential learning and professional development. While the initiative aligns with the Medical Research Council (MRC) framework for complex interventions, its primary innovation lies in its design as a scalable model for competency-based learning integrated directly into real-world practice.

**Methods:**

Following the Medical Research Council (MRC) framework, this paper reports on the first two phases: (1) concept development and (2) pilot planning. The development phase comprised a synthesis of evidence on competency-based and decentralized medical education, design of a didactic model based on Kolb’s Experiential Learning Cycle, and process/outcome modelling to guide feasibility testing. The pilot phase will assess the model’s feasibility, acceptability, and integration into routine general practice.

**Discussion:**

VISIONARA seeks to bridge the gap between theoretical academic instruction and real-world healthcare in general practice education by embedding structured, competency-based learning within authentic clinical settings. The feasibility study will examine financial and personnel resource requirements, acceptance among practice staff, students, and patients. It will further assess organizational integration into routine practice workflows, and the identification of structural and logistical barriers. These findings will inform further refinement and the potential progression to a subsequent evaluation phase.

## Zusammenfassung

**Hintergrund:**

Die Ausbildung zukünftiger Ärzt:innen zur Gestaltung eines resilienten, primärversorgungsorientierten Gesundheitssystem erfordert eine stärkere Ausrichtung der medizinischen Ausbildung auf eine dezentrale und kompetenzbasierte Ausbildung (CBME -englisch für competency-based medical education). Bislang ist die allgemeinmedizinische Ausbildung in Deutschland fragmentiert: Die theoretischen Inhalte werden an der Universität gelehrt, während in Blockpraktika in niedergelassenen Arztpraxen praktische Erfahrungen gemacht werden, in denen jedoch oft die notwendige didaktische Infrastruktur fehlt. Diese anhaltende Trennung von Theorie und Praxis verhindert einen echten Wandel hin zu einer kompetenzbasierten Ausbildung. Daher sind neue Lehrkonzepte notwendig, in denen die theoretischen Inhalte direkt in den Versorgungsalltag integriert werden.

**Ansatz:**

VISIOn Netzwerk Akademischer Lehrpraxen in der Allgemeinmedizin (VISIONARA) stellt ein neues Lehrkonzept für eine dezentraler, kompetenzbasierte Lehre in der Allgemeinmedizin vor. Aus niedergelassenen Arztpraxen werden akademische Lehrcluster, in denen strukturierte, kontextbasiere Lernumgebungen geschaffen und so erfahrungsbasiertes Lernen und Professionalisierung gefördert werden. Die Konzeptentwicklung orientiert sich an dem Medical Research Council (MRC) Framework für komplexe Interventionen. Die Innovation liegt in der Schaffung eines skalierbaren Modells für kompetenzbasiertes Lernen direkt in der Versorgungspraxis.

**Methoden:**

Orientierend an dem MRC-Framework werden die ersten beiden Phasen des Lehrclusteraufbaus beschrieben: (1) Konzeptentwicklung und (2) Planung der Pilotierung. Die Konzeptentwicklungsphase beinhaltet eine Evidenzsynthese über aktuelles Wissen zur kompetenzbasierten und dezentralen medizinischen Ausbildung und dem erfahrungsbasierten Lernen nach Kolb. Sie beinhaltet zudem eine Prozess- und Ergebnismodellierung als Vorbereitung der Pilotphase. In der Pilotphase sollen Machbarkeit, Akzeptanz und Integrationsfähigkeit in den Routinebetrieb der Arztpraxen evaluiert werden.

**Diskussion:**

VISIONARA hat den Anspruch, die bisherige Lücke von theoretischer akademischer und praktischer allgemeinmedizinischen Ausbildung durch den Aufbau eines strukturierten, kompetenzbasierten und authentischen Lehrmodells zu schließen. Die Pilotphase wird finanzielle Ressourcen, die notwendigen personellen Ressourcen, Akzeptanz des Praxispersonals, Studierender und Patienten untersuchen. Zudem wird geprüft, wie das Programm organisatorisch in den laufenden Praxisbetrieb integriert werden kann und wo strukturelle bzw. logistische Hürden liegen. Die Ergebnisse der Pilotphase werden als Grundlage von Anpassungen des Konzepts und einer potenziell nachfolgenden Evaluationsphase dienen.

## 1. Introduction

### 1.1. From knowledge acquisition to competency-based education: Theoretical foundations

Healthcare systems are undergoing structural and demographic challenges. Considering these developments, strategic efforts to ensure future-proof healthcare delivery are gaining increasing importance. A sustainable transformation of the healthcare system can only succeed if medical education is aligned with these requirements [18]. This demands a rethink in the training of our medical students: students must receive competence-based medical education (CBME) in which they acquire skills that enable them to “effectively meet the health care needs of the people they serve” [37]. 

Over the past 25 years, conceptual progress has been made to define the competencies relevant to clinical practice. Among the most widely recognized frameworks is the Canadian Medical Education Directives for Specialists (CanMEDS) Framework, which define physician roles and competencies [17], [16]. In Germany, these efforts are reflected in the development of the National Competency-Based Catalogue of Learning Objectives for Medicine (Nationaler Kompetenzbasierter Lernzielkatalog - NKLM) [29]. As part of future reforms, the NKLM is expected to inform approximately 80% of core curricula and may be integrated into the German medical licensing regulations (Ärztliche Approbationsordnung).

A core principle of CBME is that competencies cannot be reduced to the acquisition of factual knowledge. They represent abilities that emerge from the integration of knowledge, skills, and attitudes, and they only become meaningful when applied within authentic contexts of patient care. The competency-based education is inherently context-based education.

### 1.2. Implementation challenges in German medical education

Despite this clarity, the implementation of CBME in Germany remains limited. A key challenge lies in the disconnection between academic training settings and the diverse realities of healthcare delivery. At present, most medical education takes place within university facilities and teaching hospitals [36]. While these settings provide exposure to advanced technologies, rare diseases, and research-based treatments, they represent only a limited fraction of the environments in which future physicians will practice. Many essential competencies cannot be adequately developed in such centralized and institution-based environments.

The competencies that future physicians must develop are complex, encompassing not only knowledge acquisition but also the gradual formation of a professional, the ability to collaborate in interprofessional teams and to make clinical decisions under conditions of uncertainty. Crucially, this involves not only balancing measurable risks but also developing tolerance for uncertainty – learning to make responsible decisions even when probabilities are unknown and information is incomplete. Such competencies remain hidden in traditional university-based education, becoming evident only when students are exposed to uncertainty in everyday clinical decision-making and can reflect on their evolving role as future physicians [30]. 

In addition to direct clinical exposure, the facilitation of competence-based learning requires an appropriate didactic infrastructure that allows students to engage with learning content on multiple levels, integrating observation, reflection, and theoretical understanding [13], [14], [27], [52]. In those environments students move beyond theoretical knowledge to internalize professional values, assume responsibility, and develop systemic awareness – core elements of medical practice [18], [27], [45]. 

### 1.3. The current state of general practice education in Germany

This type of didactic infrastructure is limited in undergraduate primary care teaching in Germany. Mostly, students attend centrally organized courses at the university. These are supplemented by a mandatory two-week clerkship in a general practice (GP) setting, during which students gain insight into the daily routines and workflows of primary care. In addition, students are required to complete a four-week internship in a primary care setting.

On average, each medical faculty collaborates with around 170 academic teaching practices to host students during their two-week clerkship [41]. However, the practices receive only minimal financial compensation for their teaching. Despite the limited resources, many general practitioners continue to engage in student education with remarkable motivation. Their ongoing commitment is both noteworthy and commendable. Students value the clerkship for the opportunity to gain direct patient contact and to apply physical examination techniques in real-world settings. Nevertheless, the implementation of the didactic framework often lags, as teaching is provided by practitioners with limited pedagogical training who must integrate educational tasks into the demands of unpredictable daily practice.

Under current conditions there is little scope to apply structured didactic methods that would enable the kind of deep learning described above. Ensuring high-quality general practice education – and thereby preparing future physicians for resilient healthcare systems – requires a fundamental rethinking of how clinical teaching in primary care is organized, resourced, and valued. This includes recognizing decentralized, practice-based learning as a core component of medical training.

### 1.4. International comparison 

In contrast to the German system, many countries have successfully integrated decentralized teaching formats into their medical curricula [12], [32], [34], [52]. Rural and community-based curricula in these countries have demonstrated not only equivalent or better examination outcomes compared to standard programs but also increased professional identity development [4], [5], [38], [51]. Students in decentralized programs reported greater alignment between clinical theory and patient needs, increased competence in interprofessional collaboration, and a deeper understanding of healthcare systems [3], [8], [32]. 

Taken together, international evidence underscores that context-based, decentralized teaching is not only feasible but also highly effective. With this background, we introduce VISIONARA as a teaching model designed to establish sustainable, competency-oriented learning environments. The novelty of VISIONARA lies in conceptualizing decentralized primary care education as a coherent complex intervention, in which community-based training is systematically combined with additional evidence-informed didactic strategies rather than representing a mere relocation of teaching activities. The model considers both pedagogical and organizational aspects of implementation in primary care. The following section outlines the development of the model.

## 2. Methods

VISIONARA is a reference model for undergraduate general practice education that establishes decentralized teaching clusters to provide the structural and personnel framework necessary for high-quality, context-based learning. The project follows the Medical Research Council (MRC) Framework for Complex Interventions [11], [40]. The initial Framework distinguishes four key stages:

1. *Development* – identifying existing evidence, developing theory, and modelling processes and expected outcomes;
2. *Feasibility and piloting *– testing the acceptability and practicality;
3. *Evaluation* – assessing effectivity and mechanisms;
4. *Implementation* – ensuring transferability, scalability, and sustainability in real-world settings.

Across all stages, six core elements are addressed iteratively (see table 1 (Tab. 1)). This paper describes the development phase and outlines the planned feasibility phase.

### 2.1. Hypothesized long-term impact

The overarching goal of VISIONARA is to strengthen primary care–based medical education. If successfully implemented, the program is expected to contribute to:

- Establishment of sustainable educational structures in primary care through decentralized teaching clusters embedded within real-world healthcare environments
- Aligned with the NKLM, the program promotes competency-based medical education (CBME) by offering learning opportunities that develop not only factual knowledge but also professional identity and clinical reasoning skills.

### 2.2. Stage 1: Development phase

#### 2.2.1. Developing theory

An interdisciplinary expert group (faculty, health economists, practice owners, psychologists, residents, and employed GPs) met within six iterative workshops over one year. A literature review conducted by two university researchers informed the process.

Several key components were identified as essential to the design of the new education program (see figure 1 (Fig. 1)). Situated, community-based environmental learning is central, as learning cannot be separated from the context in which it occurs. The program also requires clinician teachers with dual expertise – professionals who are both clinically competent role models and didactically trained educators [28], [39]. Protected teaching time is necessary so that clinical teaching does not compete with patient care responsibilities [7]. Effective learning should be in line with Experiential Learning Theory, which integrates concrete experience, reflective observation, abstract conceptualization, and active experimentation [1], [8], [27], [50], [52]. Peer learning, in which students benefit from each other’s knowledge, feedback, and perspectives, represents an effective and well-established instructional approach [15]. Finally, the existing two-week general practice clerkship in Germany already provides valuable real-world exposure [44], making it a foundation for further program development.

#### 2.2.2. Modelling processes

Based on the expert discussions, a model for VISIONARA was developed. The mandatory two-week clerkship was selected as the initial implementation format, with the intention of expanding VISIONARA to further primary health care teaching formats if feasibility can be demonstrated.

Structural criteria for participating practices were defined to preserve authenticity, ensure that students can conduct appropriate patient consultations under supervision, and provide role models of contemporary primary care practice. Participating sites must therefore be owner-managed, interprofessional general practices (including GPs, MFAs and a physician assistants) and provide at least two consultation rooms for the students.

To transform these practices into academic teaching clusters, additional didactic capacity and time are provided. As protected teaching time may result in financial losses for practice owners, economic implications are considered according to project phase, and participating practices must be financially compensated for their teaching activities. All participating team members receive didactic training, with advanced didactic preparation for designated teaching physicians.

Instead of one-to-one teaching, groups of four clerkship students will be trained in parallel within each teaching cluster, thereby using the benefits of peer learning while also reducing the overall number of practices required for the clerkships and enabling a more resource-efficient implementation. The developed curriculum combines complementary learning formats – workplace-based teaching during routine patient care, theory seminars, structured reflection modules, observation of care settings, and e-learning – organized into thematic learning days (see figure 2 (Fig. 2)). 

### 2.3. Stage 2: Feasibility and piloting

The model will be initially implemented over a six-month period in three general practices that meet the defined structural criteria and already maintain close collaboration with the university. Stage 2 aims to assess the practical feasibility and acceptance of the VISIONARA teaching model.

#### 2.3.1. Outcome measurements

Quantitative and qualitative data will inform both comparative analyses and iterative refinement for for further model development toward a potential controlled trial.

Quantitative data will be collected post-intervention from practice staff (practice owners, teaching physicians, practice managers, medical assistants), students, and patients. Implementation outcomes will be assessed using validated instruments: the Acceptability of Intervention Measure (AIM), the Intervention Appropriateness Measure (IAM), and the Feasibility of Intervention Measure (FIM) [48]. Mean values below 3 will be interpreted as indicating critical implementation barriers. 

Qualitative data will be conducted via semi-structured individual interviews. Interviews will be audio-recorded, transcribed verbatim, and analyzed using qualitative content analysis.

- *Feasibility and acceptance (practice staff): *Pre-intervention interviews explore perceived feasibility, anticipated challenges, and training needs. Post-intervention interviews (after four student cohorts) assess implementation experiences, barriers, benefits, infrastructure, and sustainability.
- *Acceptance (students and patients):* Only Post-intervention interviews will be conducted. Students will reflect on their learning experience and integration of theory and practice. Patients will be asked about their acceptance of student involvement, potential concerns regarding participation in teaching consultations and the overall relevance and appropriateness of student participation in their care.

#### 2.3.2. Sample size justification

Sample size is based on commonly used estimates for pilot research rather than formal power calculations [43]. Assuming five practice staff members per site, this results in 15 practice stakeholders for interview and survey participation during the six-month study period. During this time, three student cohorts attend the clerkships (4 students × 3 cohorts × 3 practices = 36 students). Assuming a response rate of approximately 50%, around 18 student questionnaires are expected. Patient feedback will be collected during routine consultations. Based on consultation volumes and an anticipated response rate of about 5%, approximately 50 patient questionnaires are expected.

#### 2.3.3. Resources

Participating practices will receive financial compensation for their teaching activities. During the feasibility phase, additional costs related to the development and preparation of the teaching format must also be considered. Resource allocation specific to the feasibility phase is detailed in table 2 (Tab. 2).

## 3. Discussion

Preparing physicians to contribute to a sustainable and high-quality healthcare system requires learning environments that go beyond factual knowledge and are grounded in settings in which theoretical knowledge can be meaningfully integrated [18], [39]. In this regard, existing university-centered teaching structures are insufficient for future-oriented primary care education and require systematic redesign [9].

VISIONARA addresses this need by developing a decentralized academic teaching model that integrates several evidence-informed components shown to promote competency development into a coherent and context-sensitive concept [1], [7], [35], [39], [50]. Internationally, decentralized medical education has demonstrated both feasibility and educational benefit [4], [5], [49], [52].

In Germany, the general practice clerkship is already delivered in decentralized settings, and students value the opportunity for direct patient contact and insight into real-world care processes [44]. However, while structured pedagogical guidelines exist for teaching staff, there is still a lack of a clearly defined pedagogical concept and detailed implementation plan directly within the practices. Specifically, current approaches do not fully address the development of more complex competencies that extend beyond factual knowledge, such as professional identity formation, interprofessional collaboration, and clinical reasoning skills, and their integration with fact-based teaching remains limited.

Teaching activities often compete with routine patient care, which limits educational effectiveness [19], [46]. Moreover, increasing workload and rising patient volumes in general practice have further reduced the capacity of many practices to host students, even when physicians remain motivated to engage in teaching. VISIONARA addresses these deficits by establishing academic teaching clusters with protected teaching time, didactic preparation, and a defined curriculum. 

The forthcoming feasibility study will examine whether this model is acceptable and feasible from the perspectives of students, teaching physicians, and practice staff, and what effects it has on patient care, which could be positive but could have also compromising effects [31]. Potential barriers include travel distance and organizational challenges. Prior studies suggest that acceptance among students increases when travel expenses, accommodation, or public transport access are adequately supported [6], [23]. 

Furthermore, VISIONARA introduces a group-based learning approach with up to four students per cluster. Evidence suggests that such peer-based learning formats can enhance professional self-efficacy without reducing competency attainment compared to traditional one-on-one teaching [22]. In the long term, strengthening evidence-based clinical reasoning among graduates may have implications in healthcare quality and efficiency [2], [20]. At the current developmental stage, however, such outcomes remain theoretical projections and are not the primary focus of the feasibility evaluation.

A limitation of the current available evidence on decentralized teaching is that most studies rely on non-randomized designs and self-reporting measurements of the students [25], [30]. Similarly, our feasibility study is subject to this limitation. However, at the developmental stage – where the primary focus lies on perceived acceptability and individual feasibility within routine practice settings – this approach is methodologically appropriate. To strengthen the evidence base, future research should include external measures of competency development, such as results in Objective Structured Clinical Examinations [21] or Entrustable Professional Activities [42]. Consequently, after completion of the feasibility phase, a randomized controlled trial will be necessary to evaluate whether VISIONARA achieves its targeted competency outcomes.

The cluster model may also address the growing shortage of available teaching practices. If five clerkship cohorts complete the mandatory two-week rotation per year, a single VISIONARA cluster could supervise 20 students annually. With approximately 360 students per cohort, 18 teaching clusters would theoretically suffice to provide universal clerkship capacity. Compared with traditional one-to-one placements this represents a potentially more personnel-efficient model while maintaining educational quality. 

Economic considerations represent a critical dimension. Establishing academic teaching clusters requires substantial financial investment, and even after stabilization VISIONARA will remain more resource-intensive than the current ambulatory teaching model. This raises a policy question about the value assigned to high-quality outpatient medical education. At the same time, German healthcare policy promotes a shift from inpatient to outpatient care to strengthen ambulatory structures and reduce avoidable hospital treatment [26]. Advancing this orientation necessitates corresponding investment in robust outpatient training models. Following the feasibility phase, preliminary cost estimates will inform discussions with political stakeholders and regional authorities on financing strategies.

The aim of VISIONARA is not to replace hospital-based education but to complement it by strengthening the ambulatory component of medical training. The forthcoming feasibility study will provide essential information on acceptability, feasibility, and contextual factors, forming the basis for future evaluation and potential broader implementation. If feasible, VISIONARA may offer a structured and scalable model for integrating decentralized, competency-based teaching into general practice education in Germany.

## Authors’ ORCIDs

- Judith Lübbert: [0009-0002-3803-0777]
- Dorothee Schüle: [0009-0007-2337-2287]
- Attila Altiner: [0000-0002-2429-933X]
- Svetla Loukanova: [0000-0003-3183-414X]
- Rüdiger Leutgeb: [0000-0003-4240-0822]

## Competing interests

The authors declare that they have no competing interests. 

## Figures and Tables

**Table 1:**
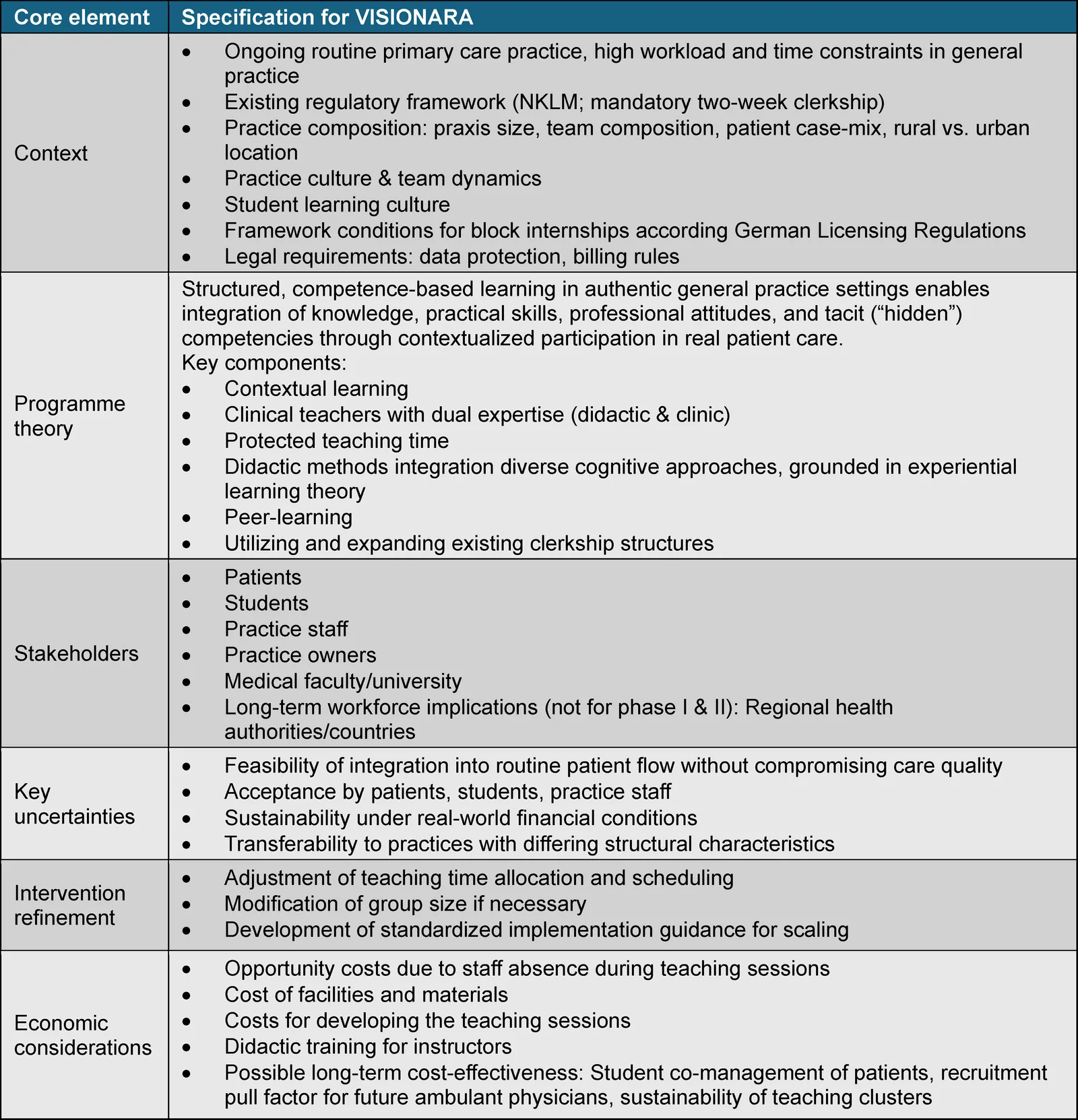
MRC Core elements of VISIONARA, based on [40]

**Table 2:**
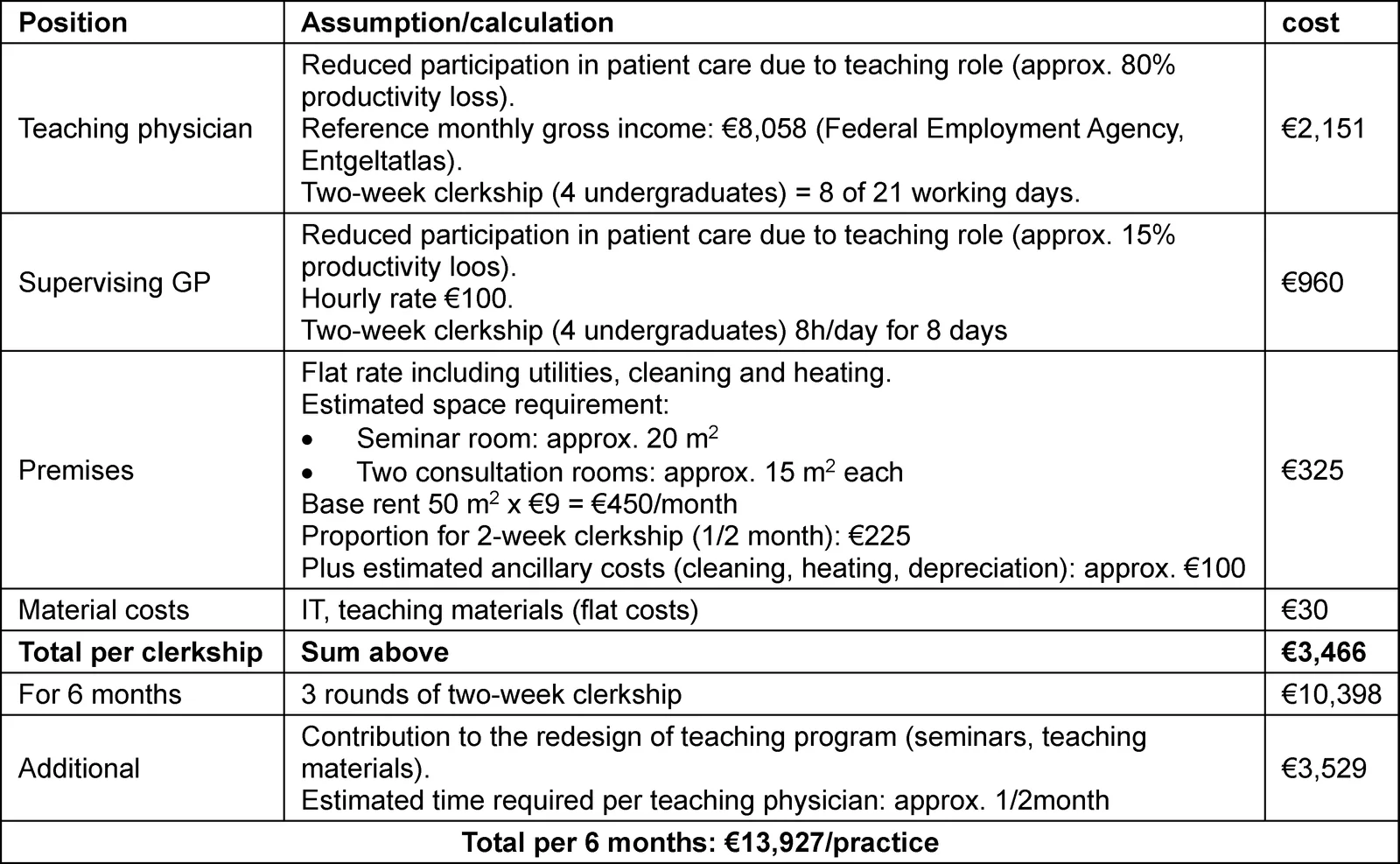
Cost calculation for the feasabilty study for each praxis

**Figure 1:**
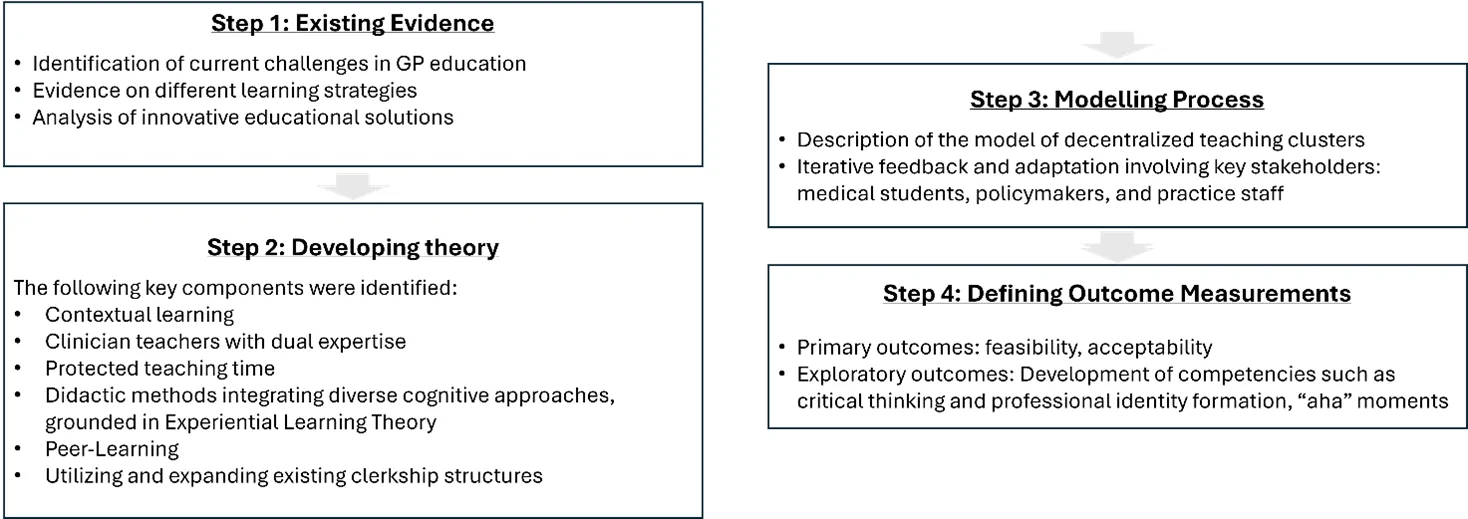
Different steps of the development phase (adapted on stage 1 of MRC framework [11], [40])

**Figure 2:**
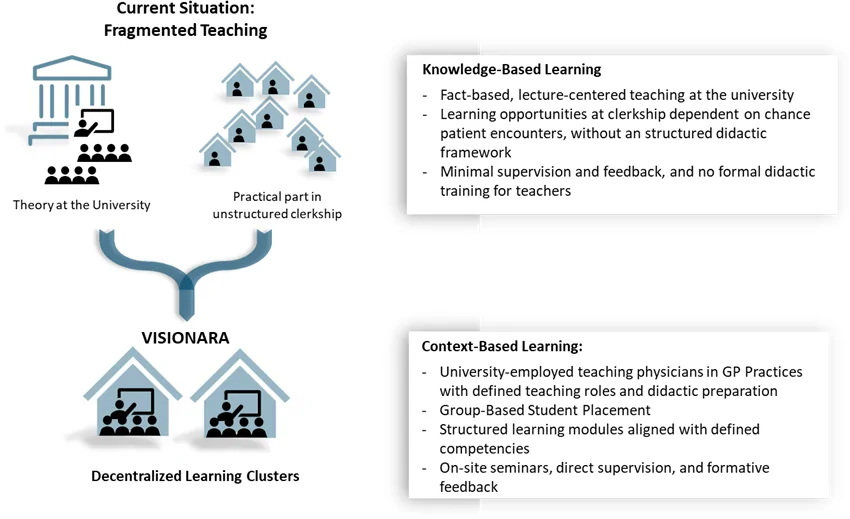
Comparison of current GP teaching structure and decentralized learning clusters
